# Supplementary material for: New Concept for the Facile Fabrication of Core–Shell CuO@CuFe2O4 Photocathodes for PEC Application
Source: Materials (Basel). 2022 Jan 28;15(3):1029. doi: 10.3390/ma15031029 (PMC8839972; doi:10.3390/ma15031029)
Supplement: Supplementary file 1 [file materials-15-01029-s001.zip › materials-1558196-supplementary.pdf]

Supplementary Materials: NEW CONCEPT FOR THE FACILE FABRICATION OF CORE-SHELL $CuO@CuFe_2O_4$ PHOTOCATHODES FOR PEC APPLICATION

Linh Trinh [1],*, Krzysztof Bienkowski [1], Piotr Wróbel [2], Marcin Pisarek [3], Aleksandra Parzuch [1], Nabila Nawaz [1], and Renata Solarska [1],*

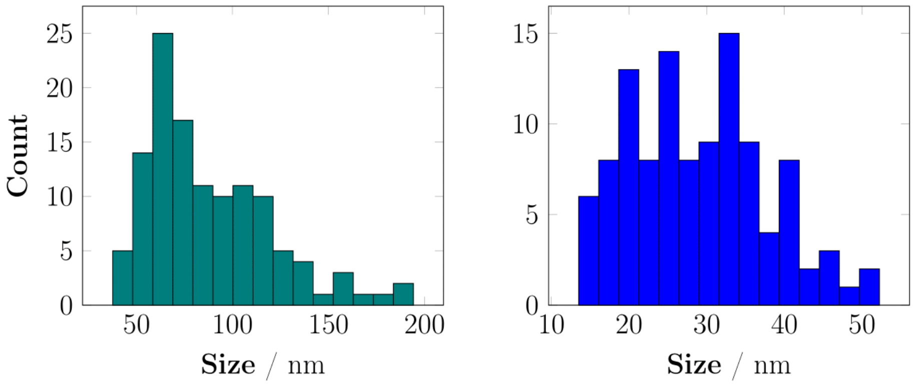

Figure S1. Size distribution of $CuFe_2O_4$ obtained from SEM imaging

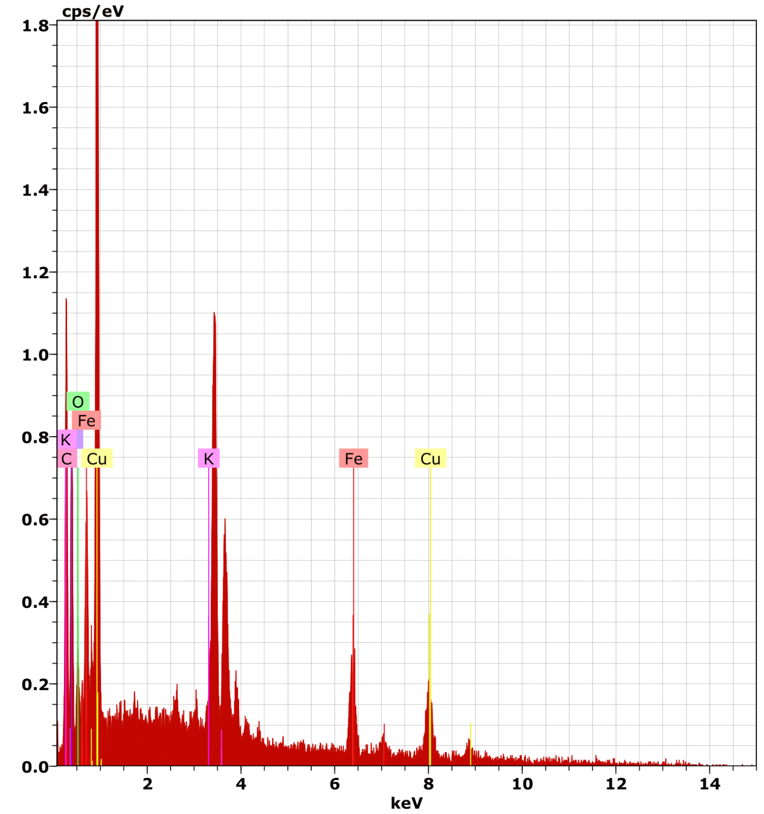

Acquisition 216 Date:1/20/2021 9:59:32 AM HV:15.0kV Puls th.:1.33kcps

El	AN	Series	unn. C [wt.%]	norm. C [wt.%]	Atom. C [at.%]	Error (1 Sigma) [wt.%]
N	7	K-series	13.18	30.57	45.15	2.51
Cu	29	K-series	12.05	27.95	9.10	0.56
C	6	K-series	7.49	17.37	29.91	1.41
Fe	26	K-series	5.84	13.55	5.02	0.26
O	8	K-series	2.95	6.84	8.85	0.79
K	19	K-series	1.61	3.73	1.97	0.10
		Total:	43.13	100.00	100.00	

Figure S2. EDS analysis of CuFe PBAs

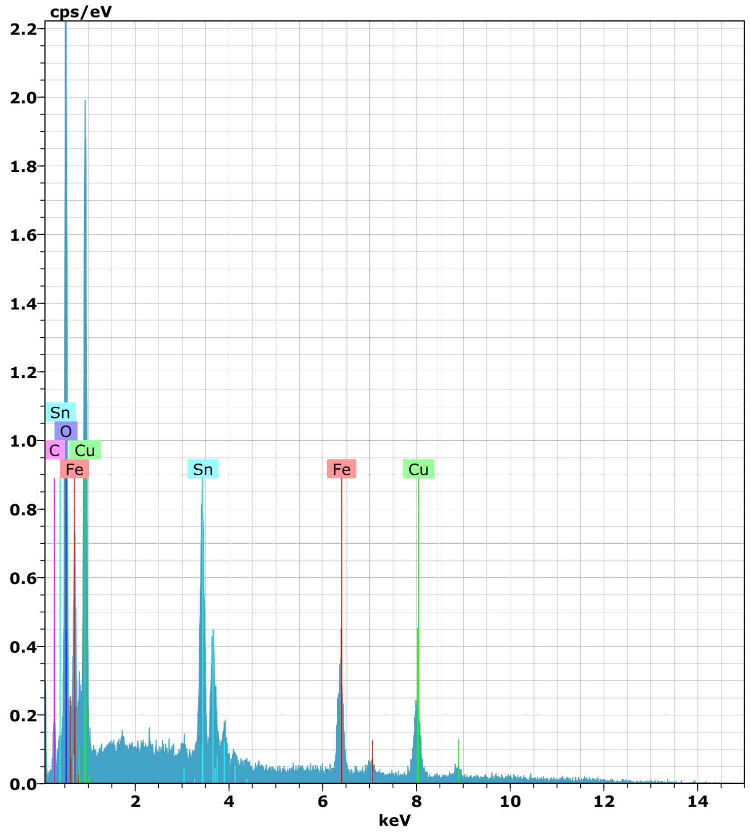

Objects 316 Date:11/5/2021 10:48:21 AM HV:15.0kV Puls th.:1.27kcps

El	AN	Series	unn. C [wt.%]	norm. C [wt.%]	Atom. C [at.%]	Error (1 Sigma) [wt.%]
O	8	K-series	25.67	29.52	61.62	3.75
Cu	29	K-series	23.17	26.64	14.00	0.97
Sn	50	L-series	21.98	25.27	7.11	0.74
Fe	26	K-series	13.68	15.73	9.41	0.53
C	6	K-series	2.46	2.83	7.86	0.77
		Total:	86.96	100.00	100.00	

Figure S3. EDS analysis of $CuO@CuFe_2O_4$

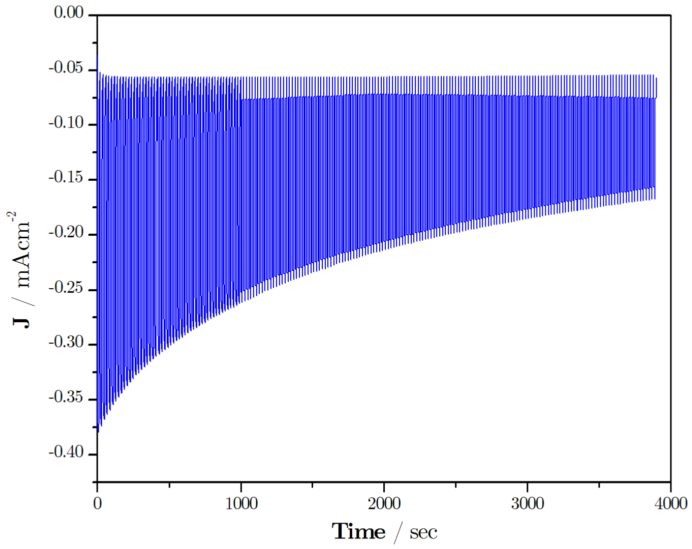

Figure S4. Extended i-T curve of CuO@$CuFe_2O_4$ in NaOH 0.1 M (pH 13) under chop light irradiation

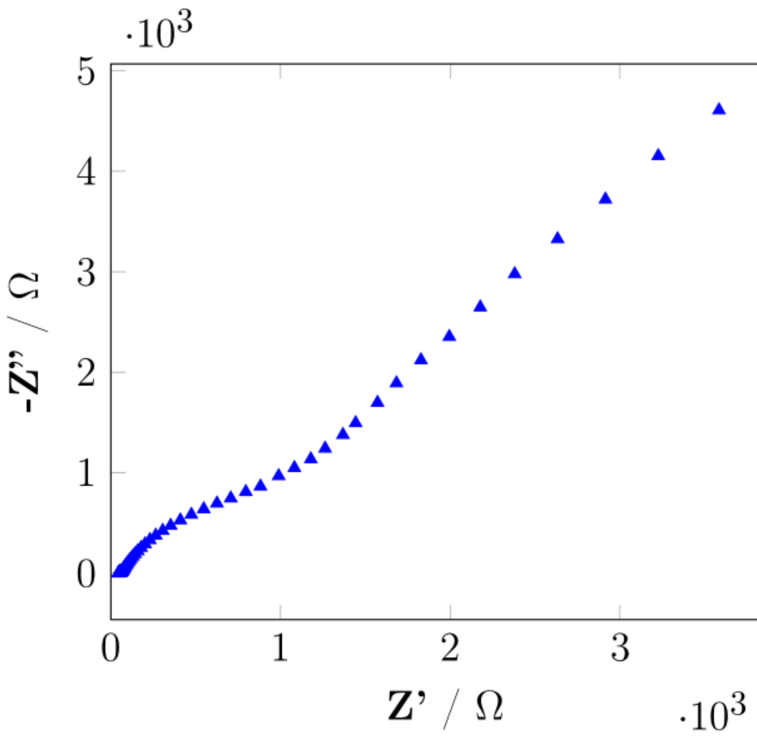

Figure S5. Nyquist measurement of CuO@$CuFe_2O_4$ in NaOH 0.1 M (pH 13) under 1 sun light irradiation

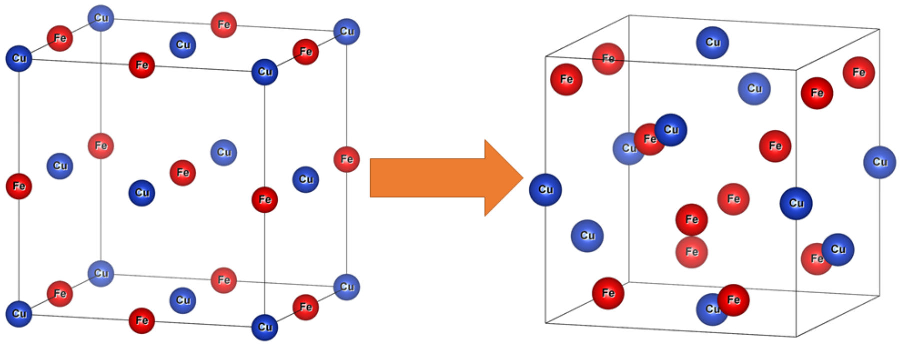

Figure S6. Crystal structure of CuFe PBAs before and after thermal conversion

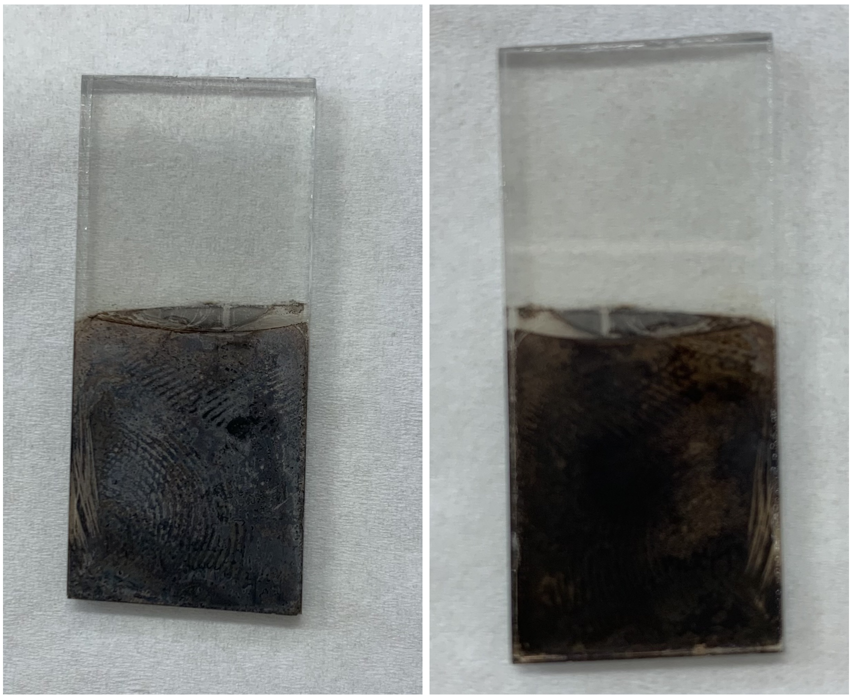

Figure S7. Images of the front and back side of prepared $CuOCuFe_2O_4$ sample
